# Vulnerability of Southern Hemisphere bats to white‐nose syndrome based on global analysis of fungal host specificity and cave temperatures

**DOI:** 10.1111/cobi.14390

**Published:** 2024-10-15

**Authors:** Nicholas C. Wu, Justin A. Welbergen, Tomás Villada‐Cadavid, Lindy F. Lumsden, Christopher Turbill

**Affiliations:** ^1^ Hawkesbury Institute for the Environment Western Sydney University Richmond New South Wales Australia; ^2^ Department of Energy, Environment and Climate Action Arthur Rylah Institute for Environmental Research Heidelberg Victoria Australia; ^3^ School of Science Western Sydney University Richmond New South Wales Australia

**Keywords:** Chiroptera, disease, hibernation, *Pseudogymnoascus destructans*, vulnerability ecological naivety, Chiroptera, enfermedad, hibernación, ingenuidad ecológica, vulnerabilidad, *Pseudogymnoascus destructans*

## Abstract

White‐nose syndrome (WNS), a disease affecting hibernating bats, is caused by the fungal pathogen *Pseudogymnoascus destructans* (Pd). Since the initial introduction of Pd from Eurasia to the United States in 2006, WNS has killed millions of bats throughout the temperate parts of North America. There is concern that if Pd is accidentally introduced to the Southern Hemisphere, WNS could pose similar threats to the bat fauna of the Southern Hemisphere's more temperate regions. Efforts are required to better understand the vulnerability of bats globally to WNS. We examined phylogenetic distances among cave roosting bat species globally to estimate the probability of infection by Pd. We predicted cave thermal suitability for Pd for 441 cave‐roosting bat species across the globe via spatial analysis. We used host specificity models based on 65 species tested for Pd to determine phylogenetic specificity of Pd. Phylogenetic distance was not an important predictor of Pd infection, confirming that Pd has low host specificity. We found extensive areas (i.e., South America, Africa, and Australia) in the Southern Hemisphere with caves that were suitable for cave‐roosting bat species and for Pd growth. Hence, if Pd spreads to the Southern Hemisphere, the risk of exposure is widespread for cave‐roosting bats, and infection is possible regardless of relatedness to infected species in the Northern Hemisphere. Predicting the consequences of infection remains difficult due to lack of species‐specific information about bat winter biology. Nevertheless, WNS is an important threat to naive Southern Hemisphere bat populations. Hence, biosecurity measures and planning of management responses that can help prevent or minimize a potential WNS outbreak in the Southern Hemisphere are urgently needed.

## Abstract (es)

Vulnerabilidad de los murciélagos del hemisferio sur ante el síndrome de nariz blanca con base en el análisis mundial de especificidad del hospedero fúngico y las temperaturas de las cuevas

**Resumen:**

El síndrome de nariz blanca (SNB), una enfermedad que afecta a los murciélagos en hibernación es causado por el hongo patógeno *Pseudogymnoascus destructans* (Pd). Desde la introducción inicial del Pd desde Eurasia a Estados Unidos en 2006, el SNB ha matado a millones de murciélagos en las zonas templadas de Norteamérica. Existe la preocupación de que, si el Pd se introduce por accidente en el hemisferio sur, el SNB podría amenazar de forma similar a los murciélagos en las regiones más templadas de este hemisferio. Se requieren esfuerzos para comprender mejor la vulnerabilidad de los murciélagos ante el SNB a nivel mundial. Analizamos las distancias filogenéticas entre las especies de murciélagos de cuevas de todo el mundo para estimar la probabilidad de infección por Pd. Mediante un análisis espacial, predijimos la idoneidad térmica de las cuevas para el Pd en 441 especies de murciélagos de todo el mundo. Usamos modelos de especificidad de hospedero basados en 65 especies analizadas para determinar la especificidad filogenética de Pd. La distancia filogenética no fue un factor predictivo importante de la infección por Pd, lo que confirma que la especificidad de hospedero de Pd es baja. En el hemisferio sur encontramos amplias zonas (América del Sur, África y Australia) con cuevas adecuadas para especies de murciélagos que se refugian en cuevas y para el crecimiento de Pd. Por lo tanto, si Pd se propaga en el hemisferio sur, el riesgo de exposición es generalizado para los murciélagos que pernoctan en cuevas, y la infección es posible independientemente del parentesco con especies infectadas en el hemisferio norte. Sigue siendo difícil predecir las consecuencias de la infección debido a la falta de información específica sobre la biología invernal de los murciélagos. No obstante, el SNB es una amenaza importante para las poblaciones de murciélagos del hemisferio sur que no están preparadas para enfrentarla. Por ello se necesiten medidas urgentes de bioseguridad y planificación de respuestas de gestión que puedan ayudar a prevenir o minimizar un posible brote del SNB en el hemisferio sur.

## INTRODUCTION

Emerging infectious diseases are a growing threat for biodiversity worldwide (Daszak et al., 2000; Jones et al., 2008; Tompkins et al., 2015), primarily due to climate change and an increase in global transportation and human movement (Altizer et al., 2013; Banks et al., 2015; Cavicchioli et al., 2019; Lafferty, 2009). Given the negative ecological and socioeconomic repercussions of emerging infectious diseases (Lafferty et al., 2015; Scheele et al., 2019), it is crucial to predict the likely exposure and sensitivity of populations to the global spread of pathogens (Daszak et al., 2000; Lips et al., 2006), particularly because novel pathogens can have severe impacts on evolutionarily naive hosts (Frick et al., 2010; Hulcr & Dunn, 2011). Attempts to make such predictions are important for identifying and prioritizing biosecurity, research, and management efforts to mitigate the risk of emerging infectious diseases before arrival, when they are most effective (Langwig et al., 2015).

White‐nose syndrome (WNS) is a disease of hibernating bats caused by the fungal pathogen *Pseudogymnoascus destructans* (Pd) and is of particular interest due to its recent invasion from Eurasia and rapid spread across North America (Frick et al., 2010; Warnecke et al., 2012). It is responsible for the death of millions of cave‐roosting insectivorous bats since its introduction in 2006 to North America; some populations have declined by >90% (Cheng et al., 2021; Frick et al., 2017). To date, Pd has not been detected in the countries sampled in the Southern Hemisphere (i.e., Australia and Chile) (Holz et al., 2018; Lilley et al., 2020), likely indicating Pd has yet to cross the equator. The accidental introduction of Pd to bat communities in the Southern Hemisphere that are naive to this pathogen could have serious ecological, evolutionary, and economic implications (Faulkner et al., 2020; Pyšek et al., 2020). This is because the Southern Hemisphere hosts a diverse range of bat species, endemism of cave‐roosting bats in the Austral‐Oceania region is high (Tanalgo et al., 2022), and some species act as agricultural pest controls (Bouarakia et al., 2023). An expert risk assessment undertaken in Australia in late 2016 concluded that it was likely that Pd would be accidently introduced into an Australian cave within the next 10 years (Holz et al., 2019), and accidental introduction is similarly likely or more likely in South America and Africa given their proximity to Pd spread by humans and migrating bats from North America and Eurasia, respectively. Therefore, determining where Pd is likely to spread and how it may affect local bat species in the Southern Hemisphere is of global conversation priority.

Predicting the vulnerability of naive bat populations to Pd infection is challenging because information on the key factors that influence their sensitivity to the pathogen is lacking. These factors include overwintering behavior and hibernation patterns, metabolic physiology, immune function, diversity of cutaneous microbiomes and their response to Pd infection, as well as knowledge about spatial distribution of cave habitats and other factors that determine exposure to Pd (Fritze et al., 2021; Jackson et al., 2022a; Moore et al., 2018; Vanderwolf et al., 2021). However, one can make some predictions of risk of exposure to Pd and possible sensitivity to developing WNS and hence work toward estimating vulnerability (Williams et al., 2008) by focusing on some more general risk factors identified by research efforts in North America and Europe. At a broad level, the risk of exposure to WNS can be predicted by whether a species roosts in caves during winter (roost preference) and whether cave temperatures and humidity would allow for Pd growth (suitability) (Marroquin et al., 2017; Verant et al., 2012). If a bat population is exposed to Pd, its sensitivity to WNS can be predicted by inference about the likelihood of infection for a given species, its response to infection (i.e., Pd load) (Langwig et al., 2016; Zukal et al., 2016), and the severity of winter and length of hibernation (hibernation biology) (Reeder et al., 2012; Verant et al., 2014). However, winter length is not always a predictor of WNS‐associated declines for some species, such as the tricolored bats (*Perimyotis subflavus*) and northern long‐eared bats (*Myotis septentrionalis*) (Gabriel et al., 2022; Loeb & Winters, 2022; Perea et al., 2024). Although not perfect, such an analysis would still be valuable given the possibility of severe effects from WNS on bat populations and the value of identifying geographic regions and populations most at risk of developing WNS before its arrival (Langwig et al., 2015).

One way to infer sensitivity to Pd infection for a species that has not yet been exposed to Pd is to examine the known responses of related species because related species are likely to share disease‐relevant traits, as observed in other taxonomic groups (Hoverman et al., 2011). Zukal et al. (2014) found that Pd infection is phylogenetically independent across 48 species of bats examined in North America and Eurasia. However, since this study, 17 additional species have been sampled for Pd, providing scope for an updated assessment on the phylogenetic vulnerability to Pd infection. Information on the likelihood of infection among bat species with varying degrees of evolutionary relatedness (i.e., host specificity) can provide the starting point for prioritizing research efforts on species sensitivity to WNS. If Pd shows low host specificity, as indicated by Zukal et al. (2014), vulnerability to WNS is determined more by other factors that influence sensitivity, such as the hibernation biology, combined with factors that affect exposure, such as roosting preference and roost environmental suitability for Pd growth. For example, the temperature‐dependent growth of Pd is used to determine the optimal growth and growth limits of this fungal pathogen where hibernating bats can be exposed (Escobar et al., 2014; Frick et al., 2022; Haase et al., 2021; Turbill & Welbergen, 2020; Verant et al., 2012). Therefore, understanding phylogeny and climate‐dependent exposure risk is a necessary component for determining vulnerability to infectious diseases in the absence of direct experimental exposure of Pd to naive bat species.

We used data from the literature to investigate the threat of WNS to naive Southern Hemisphere bats via a global assessment of cave thermal conditions and fungal host specificity. We first examined whether Pd infection is phylogenetically conserved in bats using host‐specificity models of bats tested for Pd. We then examined whether there are regions in the Southern Hemisphere with caves that would provide suitable thermal conditions for Pd if the fungus were introduced there. Finally, we used species‐specific roosting preferences and hibernation data (derived from a literature search) to estimate the potential exposure risk to WNS. We based our predictions of exposure risk to WNS on cave temperature and roost preference, and predictions of broad‐scale sensitivity to developing WNS on the number of frost days as a proxy for hibernation duration. We considered the potential risk of Pd exposure for cave‐roosting bats in the Southern Hemisphere and the implications for conservation management.

## METHODS

### IUCN and roost preference search

We extracted species‐level biological classification (family, genus, species), geographic distribution, and conservation status (least concern, near threatened, vulnerable, endangered, critically endangered, data deficient) for 1332 species from the order Chiroptera from the International Union for Conservation of Nature (IUCN) Red List of Threatened Species database (IUCN, 2022) (downloaded 16 May 2022). Because WNS develops only during winter hibernation and mostly affects cave‐roosting species, we also collected information on hibernation biology and use of cave roosts for these bat species. For cave roost, we identified species that at least sometimes roost in caves based on Tanalgo et al. (2022), who defined cave‐roosting species as those that “occur, use, roost, or hibernate in caves and subterranean habitats for any part of their life histories.” The Tanalgo et al. (2022) database, which categorizes 679 species as cave‐roosting bats, provides the most conservative estimate because it includes species that even only occasionally roost in caves. We included additional species that fit the definition of cave‐roosting used by Tanalgo et al. (2022) but were not present in their database and made further changes to their database based on the literature search described below (detailed in Appendix S1). This revised database included 713 species with records of cave‐roosting behavior. The definition of cave‐roosting that we followed from Tanalgo et al. (2022) includes species that are primarily tree‐roosting but have been observed at least once in a cave or roost in caves only in small parts of their range. For example, populations of the Australian chocolate wattled bat (*Chalinolobus morio*) can roost either in trees or in caves in parts of their range (e.g., no cave‐roosting populations in Tasmania, but roost exclusively in caves in the Nullarbor Plain). Because we were interested in bat species that primarily roost in caves during winter, we revised this list by excluding species that rarely been recorded in caves to derive a list of primarily cave‐roosting species. This resulted in a list of 441 primarily cave‐roosting species. The roost preference for many species has not been adequately surveyed and can vary by season. Therefore, the number of species at risk of exposure presented would exclude many data‐deficient species. Pd can survive in caves and mine‐type habitats even with the absence of bats. This means that even bats that rarely enter caves or only use them in certain seasons could still be exposed to and spread Pd.

Roost preference was obtained from Churchill (2008), Harvey et al. (2011), Monadjem et al. (2020), Moyers Arévalo et al. (2020), Nowack et al. (2020), and a primary literature search on Google Scholar for the terms “*species name*” and either “*roost preference*” or “*cave*.” We included species if their geographical distribution was publicly available from the IUCN. We also categorized the geographical hemisphere of occurrence for each species based on whether a species’ geographic range is either in the northern or the Southern Hemisphere or in both hemispheres. Data curation was formatted following Schwanz et al. (2022).

### Frost days

The severity of WNS also depends on the duration of hibernation by cave‐roosting bats, which in turn is influenced by the winter length (Hranac et al., 2021). To predict winter length, we used the average number of frost days per year because this variable more accurately predicts hibernation duration than other environmental factors (Hranac et al., 2021). We estimated the average number of frost days at the ground‐surface level based on an air temperature threshold of 2°C, which indicates that the temperature at the ground‐surface level is approaching 0°C (Campbell & Norman, 2000). We extracted the mean daily minimum air temperature (at 0.5′ resolution) from 1991 to 2020 from the CPC Global Unified Temperature database provided by the National Oceanic and Atmospheric Administration Physical Sciences Laboratory, Boulder, Colorado (United States) (https://psl.noaa.gov/data/gridded/data.cpc.globaltemp.html). Bats do not necessarily hibernate during frost days; however, this provides a broad indication of potential hibernation duration available.

### Hibernation studies

There have been relatively few studies of the thermal biology of bats during winter, and available data on torpor use were often difficult to interpret in terms of confirming if the species uses prolonged torpor during a season of hibernation. Consequently, we could not confidently assign the trait of hibernation. Given the importance of hibernation to understanding sensitivity to WNS, we examined research effort on winter hibernation in free‐ranging bats in the Northern and Southern Hemisphere and used these data to identify gaps in knowledge for species predicted to be exposed to Pd. To do this, a Boolean search of titles, abstracts, and keywords with the string (*bat*$) AND (*hibernation* or *torpor*) AND (*winter*) NOT (*mouse* OR *mice* OR *rat*$ OR *rodent* OR *squirrel* OR *vole*$ OR *lemming*$) NOT (*bird*$) was performed on Web of Science on 15 December 2022, resulting in 195 records. This broad search allowed us to capture different methods used to quantify hibernation in free‐ranging bats. We then performed a full‐text search of these 195 records on whether hibernation was recorded in a bat species during winter in the field and what species were recorded. An additional search on Google Scholar for the term “*bat winter hibernation*” was also performed.

Studies on laboratory‐induced torpor were excluded because of the artificial conditions in which torpor was exhibited and because laboratory studies do not provide information about the winter behavior of wild bats. From both Web of Science and Google Scholar, a total of 193 peer‐reviewed articles in English were retained. Articles were categorized by survey methods used to quantify winter hibernation, either by measuring skin temperature of free‐ranging bats or through other methods that provide information on hibernation behavior of bats in the wild (acoustic monitoring, cave survey, banding, camera trap, collection, PIT tagging). We measured skin temperature in the field because it provided information on hibernation length, torpor bout duration, number of arousal periods, and the thermoregulatory scope (difference between euthermic body temperature and torpor body temperature) under natural conditions (Geiser, 2021)—all important components of sensitivity to WNS (Jackson et al., 2022a, 2022b; Reeder et al., 2012). Articles were also classified as Northern Hemisphere or Southern Hemisphere based on the country where the study was conducted.

### Pd infection search

To test the host specificity of bats to Pd infection, a Boolean search using the string [(bat* OR microbat* OR *Chiroptera*) AND (*white‐nose syndrome* OR *Pseudogymnoascus destructans* OR *Geomyces destructans*)] was performed on Web of Science (from 1 January 2007 to 15 March 2022), resulting in 661 records. Title and abstract screening of the comprehensive search was conducted in Rayyan (Ouzzani et al., 2016). Additional species were searched for on the WNS website (https://whitenosesyndrome.org/static‐page/bats‐affected‐by‐wns). We collected information on whether bats were tested for Pd and whether mean Pd load (per unit of area) was determined via polymerase chain reaction.

### Data analyses

**FIGURE 1:**
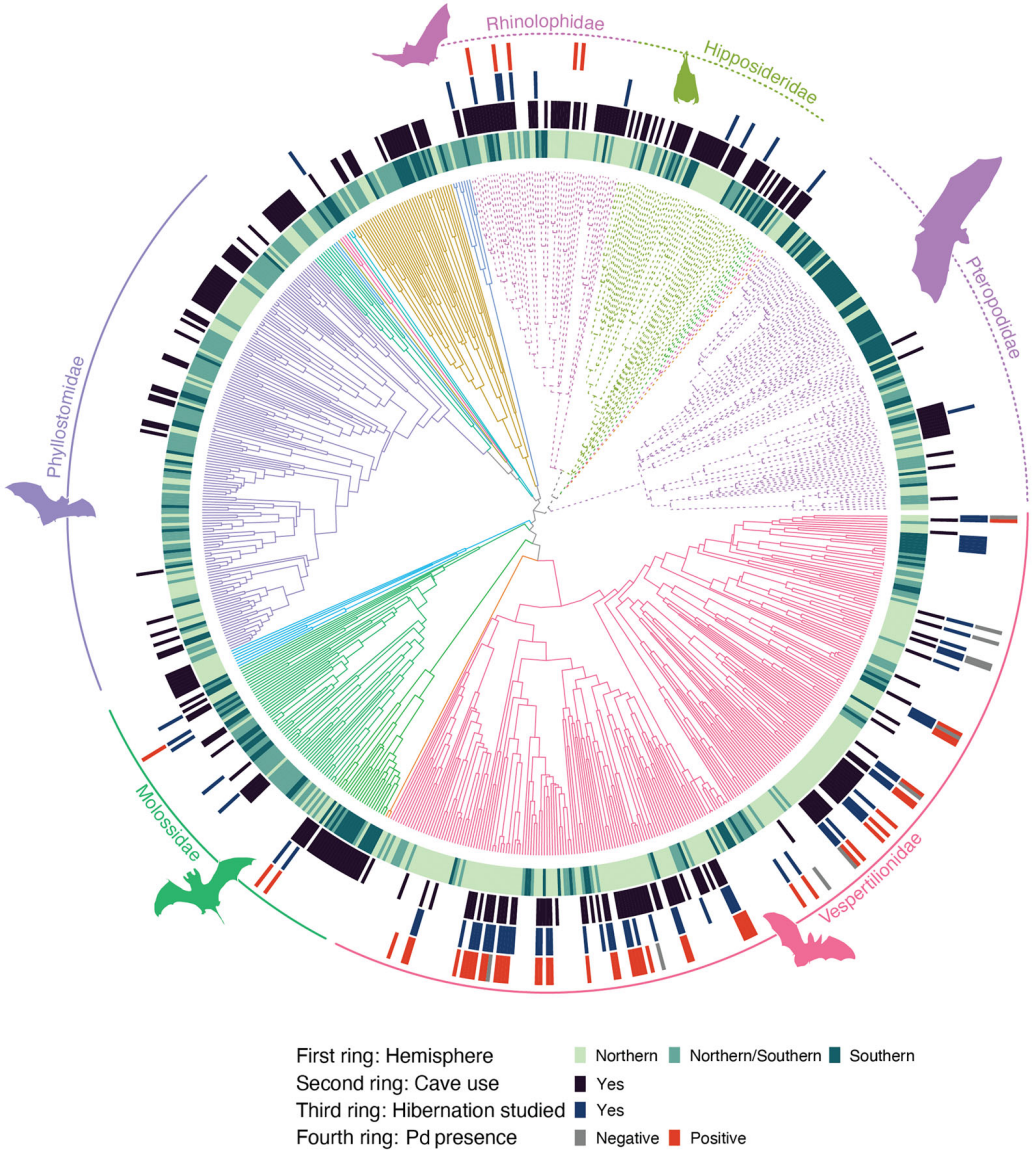
Phylogenetic reconstruction of 782 bat species from Shi and Rabosky (2015) (branches, color grouped by family; solid branches, suborder Yangochiroptera; dashed branches, suborder Yinpterochiroptera; inner first ring, hemisphere in which bats occur; second ring, whether species roosts primarily in caves; third ring, whether winter hibernation has been studied for this species in the wild; outer forth ring, whether the species has been tested and recorded as positive for Pd [red] or as negative for Pd [gray]; outer lines circling tree, 6 most diverse bat families). Bat silhouettes for families obtained from PhyloPic 2.0 (https://www.phylopic.org/).

We created 2 subsets from the full dataset (combined IUCN, hibernation, and Pd dataset): species examined for Pd (*n* = 65) and whether they developed WNS (*n* = 53). Next, we computed the pairwise distance (matrix **D**) between pairs of tips from the bat phylogeny based on its branch length with the cophenetic.phylo function from the ape package (Paradis & Schliep, 2018). We then transformed the matrix to log base 10 following Gilbert et al. (2012). We used a time‐calibrated, species‐level phylogeny from Shi and Rabosky (2015) that consisted of 812 extant species of bats (62.5% of current bat diversity estimates based on the IUCN Red List of Threatened Species database) that we organized according to species‐specific data on hemisphere region, roost preference, whether they had been studied for winter hibernation, and whether they were tested for Pd (Figure 1). Only 783 species used by Shi and Rabosky (2015) matched the bat species listed on the IUCN Red List of Threatened Species database. We then generated an incidence matrix (matrix **I**) coding a species that had tested positive or negative for Pd with the get incidence matrix function from the geotax R package (https://github.com/alrobles/geotax).

To estimate the probability that a species would be detected with Pd and the probability of a species to develop WNS, we calculated the logistic regression relating the bat Pd or bat WNS indices, respectively, in the matrix **I** to the host phylogenetic distance in matrix **D** with phylogenetic distance as the independent variable via the log reg boostrap function from geotax. A matrix of probabilities, **P**, was then generated by applying the regression coefficients to the logistic transformation of matrix **I**. The mean intercept and slope coefficient of the regression were obtained by repeating the previous procedure 1000 times. Phylogenetic signal as Pagels lambda (λ) was also quantified for mean Pd load on each bat species using the phylosig function from the phytools *R* package, with 1000 simulation replicates (Revell, 2012). We also conducted a host specificity analysis on probability of a species to develop WNS and the estimated phylogenetic signal of mean Pd load for Eurasian bats only because the outcomes could be influenced by geographical bias (North America vs. Eurasia).

We obtained spatial data of geographic distribution for 439 cave‐roosting bat species from the IUCN Red List of Threatened Species database (at 0.25′ resolution grid cell) and calculated the sum of cave‐roosting bat species in each grid cell using the calcSR function from the rasterSp R package (https://github.com/RS‐eco/rasterSp). We added 2 additional species (*Rhinolophus robertsi*, *Miniopterus orianae*) from Australia not available on the IUCN Red List using distribution ranges provided by local experts in the BatMap dataset (https://www.ausbats.org.au/batmap.html).

To estimate the maximum possible spatial extent of exposure to Pd, we mapped the geographic distribution of thermal conditions between 0 and 19.8°C allowing growth of Pd (Verant et al., 2012). We used extrapolated spatial data on mean annual surface temperature (MAST) from WorldClim 2.0 (Fick & Hijmans, 2017) as a proxy for cave temperature at a given location (Blejwas et al., 2021; Lecoq et al., 2017; Leivers et al., 2019; Perry, 2013; Vanderwolf & McAlpine, 2021). We also generated the temperature‐dependent risk map that included a formula for adjusting cave temperature based on MAST according to distance from the cave entrance because during winter cave temperature can be cooler than MAST closer to the cave entrance. A regression model was used based on McClure et al. (2020) and included an interaction between the effects of MAST and distance to the cave entrance, and predictions were validated against the observed winter cave temperatures from the literature and McClure et al. (2020) (Appendix S2). We modeled cave distances between 50 and 100 m from the entrance because 50 m is considered the start of the dark zone where bats might prefer to roost in winter (Vanderwolf & McAlpine, 2021), but even colder cave temperatures could be available if bats roost closer to the cave entrance (which potentially would result in an even larger area of suitability for Pd growth).

We then intersected the global spatial layer for richness of cave‐roosting species against areas of the MAST and cave depth‐adjusted MAST layers that predicted winter cave temperatures from 0 to 19.8°C to visualize regions of high numbers of cave‐roosting bat species in areas with the potential for Pd growth. The upper thermal limit for Pd growth is estimated as 19.0–19.8°C across all strains tested (Verant et al., 2012); we used 19.8°C as a conservative upper limit for Pd growth. Finally, we calculated the percentage of species‐level range overlap with Pd growth for cave‐roosting species in the Southern Hemisphere to predict which species are at risk of Pd exposure. We excluded Pteropodidae and Mormoopidae families because they are not known to hibernate under laboratory or wild settings (Stawski et al., 2014). Data and *R* codes are publicly available in the GitHub repository: www.github.com/nicholaswunz/WNS‐global

## RESULTS

### Diversity of cave‐roosting bats

Of the 441 species that primarily roost in caves, 247 occur in the Northern Hemisphere, 127 in the Southern Hemisphere, and 67 in both hemispheres.

### Host specificity and pathogen load

**FIGURE 2:**
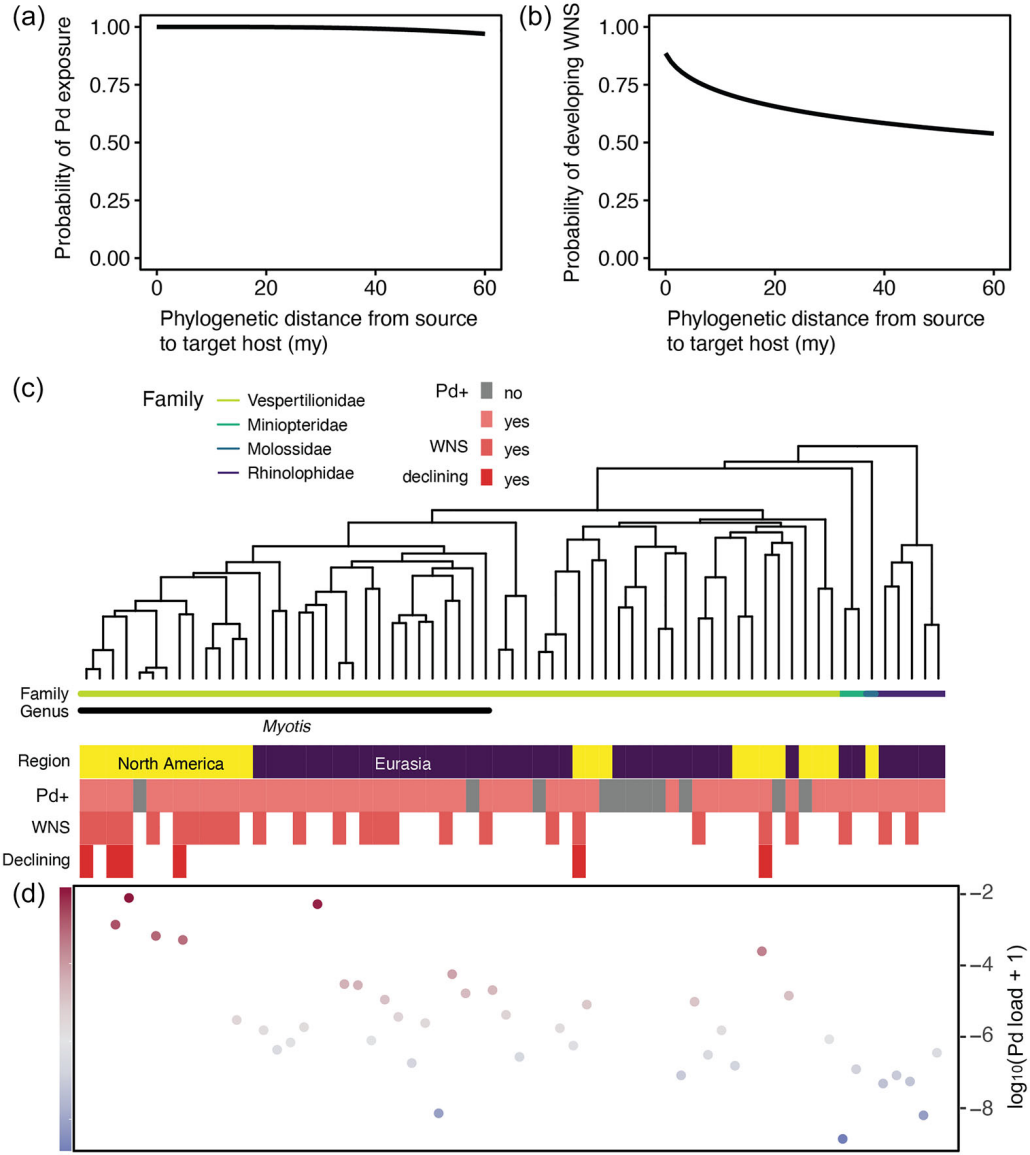
(a) Probability of host bat species being detected with *Pseudogymnoascus destructans* (Pd) based on current sampling effort in North America and Eurasia, where Pd occurs, as a function of phylogenetic distance between species, based on the 65 species that have been tested to date (curved line, main effect predicted from logistic regressions with coefficients in millions of years [my]); (b) probability of host bat species developing white‐nose syndrome (WNS) as a function of phylogenetic distance between species; (c) phylogenetic relationship of species tested for Pd in Eurasia and North America (yellow, species from North America; purple, species from Eurasia; pink, species detected with Pd; gray, species negative for Pd; dark pink, species known to develop WNS; red, species with known populations declining from WNS); and (d) base 10 logarithm of mean Pd load (ng/mm) sampled from bat species (color gradient represents Pd load intensity).

Of the 65 species tested for Pd, the likelihood of detection of Pd across the bat phylogeny (intercept = 15.37, slope = −6.83, *p* = 0.56) (Figure 2a) and the likelihood of developing WNS (intercept = 2, slope = −1.1, *p* = 0.54) (Figure 2b) showed low host specificity. However, *Myotis* species were more likely to show WNS‐associated declines (Figure 2c). This is reflected in the phylogenetic relatedness (Pagels λ = 0.90, *p* = 0.0003), where the mean Pd load was higher for *Myotis* than for species sampled from *Miniopterus*, *Rhinolophus*, *Hypsugo*, *Corynorhinus*, *Murina*, *Eptesicus*, and *Plecotus* (Figure 2d). These results should be treated with caution because sampling efforts were biased toward the *Myotis* genus (48.4% of species sampled for Pd) and the correlation between phylogeny and geography (North America vs. Eurasia) may confound the results given that closely related species are typically found on the same continent. However, a sensitivity analysis conducted on bats from just Eurasia showed low host specificity for developing WNS (intercept = 2.7, slope = −1.24, *p* = 0.53) (Appendix S3) and a moderate phylogenetic signal for mean Pd load (Pagels λ = 0.58, *p* = 0.016).

### Pd exposure

**FIGURE 3:**
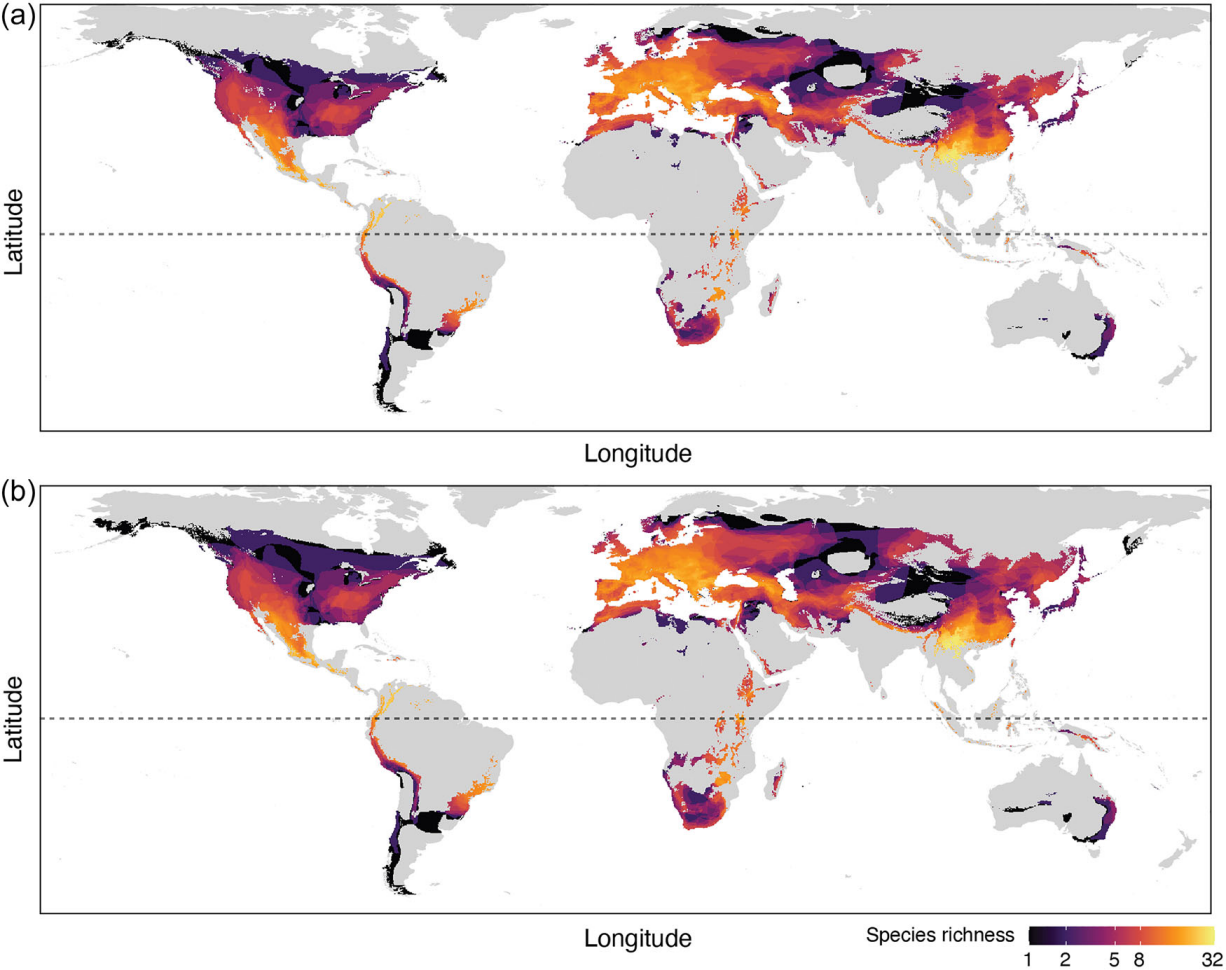
Spatial distribution of cave‐roosting bat species richness in areas with thermal conditions suitable for growth of the fungal pathogen *Pseudogymnoascus destructans* (Pd), which causes white‐nose syndrome, based on mean annual (a) surface temperature or (b) surface temperature adjusted for effect of distance to the cave entrance (50–100 m) (horizontal dashed line, equator).

Based on MAST and cave depth‐adjusted MAST from 50 to 100 m as a proxy for winter cave temperatures, there were caves suitable for Pd growth in the ranges of cave‐roosting bat species that occur in southern regions of South America, Africa, and Australia (Figure 3a,b). These regions thermally suitable for Pd growth in the Southern Hemisphere also contained numerous karst regions likely to provide deep caves (Appendix S4), but cave‐roosting bats also roosted in disused mines and culverts, which extend the thermally suitable regions of hibernacula beyond karst regions alone. Some regions in the Southern Hemisphere had >100 frost days per year, particularly in southern South America (Appendix S5). As an index of the duration of winter and the period when hibernation is required, this indicated that some bat species in regions of predicted Pd exposure could exhibit a substantial period of winter hibernation.

Of the Southern Hemisphere bats that roost in caves, there were 89 species with >5% overlap in their geographic distribution with regions thermally suitable for Pd growth (both MAST and cave depth‐adjusted MAST [list in Appendix S6]). There were 46 species predicted to have some parts of their range at risk of Pd exposure in Africa, 28 species in South America, and 15 species in the Oceania region. Most of these species with some overlap with Pd exposure risk had little‐to‐no information on their hibernation patterns (Appendix S6). Published studies on hibernation by free‐ranging bats increased by 80% since 2000 (Appendix S7), but studies in the Southern Hemisphere comprised only 10.4% of this research relative to the Northern Hemisphere.

## DISCUSSION

Novel pathogens can have catastrophic impacts on wildlife, as shown by the devastating effect of Pd on naive bat populations in North America (Cheng et al., 2021; Hoyt et al., 2021). Despite the risk that Pd could spread into Southern Hemisphere regions, there is little awareness of the likelihood of Pd infection and the potential severity of a WNS outbreak in these regions. We found that cave‐roosting bats in the Southern Hemisphere are likely to be at risk of Pd exposure due to low host specificity of developing WNS and the widespread suitable environmental temperatures in caves for Pd growth. Our study also highlighted how little is known about the winter biology of bats in Southern Hemisphere regions, which hinders more accurate predictions of their sensitivity to WNS if exposed to Pd. The range of Pd exposure in North America has, until recently, been limited to temperate regions; consequently, there are few examples to inform predictions of the impacts of WNS among naive bat species in similar climates in the Southern Hemisphere. Hence, the global significance of assessing the potential spread and impact of Pd on bat species in the Southern Hemisphere makes it a matter of considerable concern.

Previous studies have attempted to quantify exposure risk in the Southern Hemisphere at the continental scale (Escobar et al., 2014; Turbill & Welbergen, 2020), but ours is the first assessment of exposure risk with some attempt to also predict sensitivity (e.g., roosting preference, phylogeny) for cave‐roosting bats in the entire Southern Hemisphere. We identified considerable areas of exposure for cave‐roosting bats across southern parts of South America, Africa, and Australia. Our spatial analysis showed that the low‐latitude region acted as a thermal barrier to Pd crossing naturally into the Southern Hemisphere. However, with the growing unintended transportation of pathogens globally (Tatem et al., 2006) and the resilience of fungal spores carried by fomites, Pd could readily cross the equator and so represents an important emerging threat to bat fauna worldwide. Spatial information on the risk of exposure for bats is a first step to understanding their vulnerability to WNS and can help establish targeted research efforts and mitigation planning before Pd might arrive in the Southern Hemisphere.

Most cave‐roosting bat species in the Southern Hemisphere with distributions overlapping with the temperature‐dependent growth of Pd did not have information on their hibernation biology or winter energetics. This is important because of the low host specificity of developing WNS, meaning any species can potentially develop WNS. The correlation of Pd load between related species may suggest similar ecology in related species that influences WNS susceptibility, such as wintering energetics. Research from North America indicates a number of biological factors such as hibernation behavior and overwinter energetics are key predictors of species sensitivity to developing WNS (Cheng et al., 2019; Hayman et al., 2016; Jackson et al., 2022a; Moore et al., 2018). Many cave‐roosting bats use torpor to conserve energy during winter when food resources are low (Ruf & Geiser, 2015). The amount of fat stored prior to winter is closely linked to torpor and arousal patterns (Humphries et al., 2002; Kunz et al., 1998), and Pd is known to increase winter energy expenditure through increased torpor metabolic rate and an increase in the frequency and duration of interbout arousal periods (Lilley et al., 2017; Mayberry et al., 2018; Verant et al., 2014). Therefore, predicting sensitivity to Pd during hibernation for naive species requires information on 3 key factors: the amount of fat stored before winter, torpor and arousal patterns that determine energy expenditure during winter, and duration of hibernation (Hranac et al., 2021; Jackson et al., 2022b). Due to the paucity of studies describing hibernation for Southern Hemisphere bats, understanding of their sensitivity to Pd infection would benefit from research focused on the winter biology of species shown here to be most at risk of pathogen exposure.

We highlight that although the winter lengths for countries in the Southern Hemisphere are generally shorter than countries in the Northern Hemisphere (Appendix S5), suggesting that Southern Hemisphere bats are at lower risk of Pd exposure, there are uncertainties in host–pathogen dynamics that drive Pd sensitivity beyond winter length alone. Evidence of Pd exposure for cave‐roosting bats in subtropical regions of North America (https://whitenosesyndrome.org/where‐is‐wns) suggests that bats in mild climates (e.g., parts of Southern Hemisphere) might be more at risk than previously anticipated. To our knowledge, only 2 countries in the Southern Hemisphere, Australia and Chile, have tested for Pd (Holz et al., 2018; Lilley et al., 2020). Further surveillance efforts are required to detect the possible invasion of Pd in the Southern Hemisphere. Preemptive biosecurity measures should also be considered for Southern Hemisphere caves, and data could be collected to better understand the probability of entry of cave fungal spores into caves via different mechanisms (e.g., human visitation, abundance and diversity of bats in the cave, and the connectivity of cave systems) for better targeting of biosecurity measures and increased confidence in models of risk. There are notable regions with high species richness in the Southern Hemisphere where targeted research efforts are recommended. This includes most of the Andean Mountain Range, southeastern Brazil, southern South Africa, the mountainous regions of eastern Africa, Madagascar, southeastern Australia, and the mountainous regions of Papua New Guinea.

Furthermore, there is a need to establish baseline population monitoring of bats in Southern Hemisphere regions, particularly for cave‐roosting species identified as most likely to be at risk of developing WNS to determine how population numbers change if Pd is introduced. Species in the families Miniopteridae, Rhinolophidae, and Vespertilionidae are known to develop WNS in the Northern Hemisphere, and 12 species in the family Miniopteridae, 18 species in the family Rhinolophidae, and 9 species in the family Vespertilionidae found in the Southern Hemisphere may be at risk of developing WNS if exposed. There are also 2 families, Cistugidae (2 species) and Furipteridae (1 species), that are only found in the Southern Hemisphere. There is little knowledge on the wintering biology of species in these families, and their risk of developing WNS is uncertain and requires further research. Finally, several threatened species (based on the IUCN Red List) should be of high research priority because when bats are affected by other threats in addition to WNS, further declines may result. These species include *Rhinolophus cohenae* (vulnerable), *Rhinolophus smithersi* (near threatened), and *Rhinolophus ruwenzorii* (endangered) from the African continent and *Mormopterus phrudus* (vulnerable), *Platalina genovensium* (near threatened), *Tomopeas ravus* (endangered), and *Amorphochilus schnablii* (Vulnerable) in South America. However, many species listed as least concern and data deficient (Appendix S6) should also be of research interest in the context of WNS risk.

Given the increased risk of emerging pathogens globally (Jones et al., 2008; Scheele et al., 2019) and their impact on wildlife (Hoyt et al., 2021; Wu, 2023), predicting where and how they will affect naive ecosystems is an important One Health objective (Cunningham et al., 2017; Daszak et al., 2000), even if based on limited information. For example, Pd‐infected bats have increased viral load of coronaviruses (Davy et al., 2018). A Pd invasion of the Southern Hemisphere may also have potential risks for human public health given the concerns of rising zoonotic diseases (Ruiz‐Aravena et al., 2022). Such work can direct research and management efforts, which can be most effective in reducing negative outcomes from emerging wildlife diseases when initiated as early as possible (Skerratt et al., 2016; Smith et al., 2009). There is clearly a potential for spread of Pd to regions in the Southern Hemisphere, where thermal conditions are suitable for Pd growth. Even if Southern Hemisphere bat species are phylogenetically distant from Northern Hemisphere species, they are likely to be sensitive to infection, although limited information about bat winter biology hampers predictions of sensitivity to the WNS disease. Nevertheless, we advocate for a proactive response to increase targeted research efforts and instigate biosecurity and management actions that reduce the risk of entry of Pd and potentially help limit potential impacts of WNS on bats in the Southern Hemisphere.

## AUTHOR CONTRIBUTIONS

Nicholas C. Wu collected and analyzed the data, produced the figures, and wrote the initial draft. All authors contributed to conceiving the study and to the revisions.

## Supporting information



Supporting information
