# Supplementary material for: Vulnerability of Southern Hemisphere bats to white‐nose syndrome based on global analysis of fungal host specificity and cave temperatures
Source: Conserv Biol. 2024 Oct 15;39(2):e14390. doi: 10.1111/cobi.14390 (PMC11959316; doi:10.1111/cobi.14390)
Supplement: Supplementary file 1 — Supporting information [file COBI-39-e14390-s001.docx]

# SUPPLEMENTARY INFORMATION

## Corrections from Tanalgo *et al.* 2022 data

Records of cave roost data were obtained from Appendix 1 of Tanalgo et al. (2022) and cross-checked with our database. The following species from Appendix 1 of Tanalgo et al. (2022) were corrected where threatened status was based on the International Union for Conservation of Nature (IUCN) Red List:

- *Austronomus australis*: corrected “family” column from Vespertilionidae to Molossidae, removed from “cave_use” because they are not known to use caves (Richards, 1995; Churchill, 2008).
- *Chalinolobus dwyeri*: corrected “risk” column from Near Threatened to Vulnerable.
- *Chalinolobus nigrogriseus*: removed from “cave_use” because no record available on cave use (Churchill, 2008).
- *Chalinolobus tuberculatus*: corrected “risk” column from Vulnerable to Critically Endangered.
- *Chilonatalus micropus*: corrected “risk” column from Near Threatened to Vulnerable.
- *Cynopterus sphinx*: corrected “species_iucn” column from *Cynnopterus sphinx* to *Cynopterus sphinx*.
- *Emballonura serii*: corrected “risk” column from Least Concern to Vulnerable.
- *Eonycteris major*: corrected “risk” column from Data Deficient to Near Threatened.
- *Eonycteris robusta*: corrected “risk” column from Near Threatened to Vulnerable.
- *Eptesicus brasiliensis*: removed from “cave_use” because they are not known to use caves (Bredt et al., 1999).
- *Gardnerycteris crenulatum*: corrected “species_iucn” column from *Mimon crenulatum* to *Gardnerycteris crenulatum,* removed from “cave_use” because no record available on cave use.
- *Haplonycteris fischeri*: removed from “cave_use” because no record available on cave use.
- *Harpyionycteris celebensis*: corrected “risk” column from Vulnerable to Near Threatened.
- *Hipposideros curtus*: corrected “risk” column from Vulnerable to Endangered.
- *Hipposideros demissus*: corrected “species_iucn” column from *Hipposideros demissus* to *Hipposideros demissus*, corrected “risk” column from Vulnerable to Endangered.
- *Hipposideros dinops*: corrected “risk” column from Data Deficient to Vulnerable.
- *Hipposideros pomona*: corrected “risk” column from Least Concern to Endangered.
- *Hipposideros sorenseni*: corrected “risk” column from Vulnerable to Endangered.
- *Hipposideros stenotis*: corrected “risk” column from Least Concern to Vulnerable.
- *Hipposideros turpis*: corrected “risk” column from Near Threatened to Endangered.
- *Hypsugo affinis*: corrected “species_iucn” column from *Falsistrellus affinis* to *Hypsugo affinis*.
- *Hypsugo cadornae*: corrected “species_iucn” column from *Pipistrellus cadornae* to *Hypsugo cadornae.*
- *Ia io*: corrected “risk” column from Least Concern to Near Threatened.
- *Kerivoula papillosa*: removed from “cave_use” because they are not known to use caves (Seltmann et al., 2017).
- *Kerivoula pellucida*: removed from “cave_use” because no record available on cave use.
- *Kerivoula picta*: corrected “risk” column from Least Concern to Near Threatened, removed from “cave_use” because no record available on cave use.
- *Kerivoula whiteheadi*: removed from “cave_use” because no record available on cave use.
- *Lasiurus minor*: removed from “cave_use” because no record available on cave use.
- *Lissonycteris angolensis*: corrected “species_iucn” column from *Myonycteris angolensis* to *Lissonycteris angolensis*.
- *Lonchophylla dekeyseri*: corrected “species_iucn” column from *Lonchophylla dekeyserii* to *Lonchophylla dekeyseri*.
- *Miniopterus fuliginosus*: corrected “risk” column from Data Deficient to Least Concern.
- *Miniopterus macrocneme*: corrected “risk” column from Data Deficient to Least Concern.
- *Miniopterus pallidus*: corrected “risk” column from Data Deficient to Near Threatened.
- *Miniopterus schreibersii*: corrected “risk” column from Near Threatened to Vulnerable.
- *Mormoops blainvillei*: corrected “species_iucn” column from *Mormoops blainvillii* to *Mormoops blainvillei*.
- *Murina aurata*: corrected “risk” column from Least Concern to Data Deficient, removed from “cave_use” because they are not known to use caves (Smith et al., 2010).
- *Murina chrysochaetes*: removed from “cave_use” because no record available on cave use.
- *Murina eleryi*: corrected “risk” column from Data Deficient to Least Concern.
- *Murina huttoni*: removed from “cave_use” because they are not known to use caves (Molur et al., 2002).
- *Murina puta*: corrected “risk” column from Near Threatened to Least Concern.
- *Murina tenebrosa*: corrected “risk” column from Endangered to Critically Endangered.
- *Myotis formosus*: corrected “risk” column from Least Concern to Near Threatened.
- *Myotis frater*: corrected “risk” column from Data Deficient to Least Concern.
- *Myotis grisescens*: corrected “risk” column from Near Threatened to Vulnerable.
- *Myotis lucifugus*: corrected “risk” column from Least Concern to Endangered.
- *Myotis leibii*: corrected “risk” column from Least Concern to Endangered.
- *Myotis macrotarsus*: corrected “risk” column from Near Threatened to Least Concern.
- *Myotis montivagus*: corrected “risk” column from Least Concern to Data Deficient.
- *Myotis petax*: corrected “risk” column from Data Deficient to Least Concern.
- *Myotis pilosus*: corrected “risk” column from Near Threatened to Vulnerable.
- *Myotis rufoniger*: corrected “risk” column from Data Deficient to Least Concern.
- *Myotis septentrionalis*: corrected “risk” column from Least Concern to Near Threatened.
- *Myotis stalkeri*: corrected “risk” column from Data Deficient to Least Concern.
- *Notopteris neocaledonica*: corrected “risk” column from Vulnerable to Endangered.
- *Nyctalus aviator*: removed from “cave_use” because no record available on cave use.
- *Nyctophilus bifax*: removed from “cave_use” because no record available on cave use.
- *Ozimops beccarii*: corrected “species_iucn” column from *Mormopterus beccarii*to *Ozimops beccarii*.
- *Ozimops loriae*: corrected “species_iucn” column from *Mormopterus loriae* to *Ozimops loriae*.
- *Parastrellus hesperus*: corrected “species_iucn” column from *Pipistrellus hesperus* to *Parastrellus hesperus*.
- *Phoniscus papuensis*: corrected “risk” column from Least Concern to Vulnerable.
- *Platyrrhinus ismaeli*: corrected “risk” column from Data Deficient to Near Threatened.
- *Plecotus austriacus*: corrected “risk” column from Least Concern to Near Threatened.
- *Ptenochirus minor*: removed from “cave_use” because no record available on cave use.
- *Rhinolophus arcuatus*: corrected “risk” column from Least Concern to Data Deficient.
- *Rhinolophus formosae*: corrected “risk” column from Near Threatened to Least Concern.
- *Rhinolophus guineensis*: corrected “risk” column from Vulnerable to Endangered.
- *Rhinolophus ruwenzorii*: corrected “risk” column from Vulnerable to Endangered.
- *Rhinolophus osgoodi*: corrected “risk” column from Data Deficient to Least Concern.
- *Rhinolophus rex*: corrected “risk” column from Least Concern to Endangered.
- *Rhinolophus shortridgei*: corrected “risk” column from Least Concern to Data Deficient.
- *Rousettus leschenaultii*: corrected “risk” column from Least Concern to Near Threatened.
- *Rousettus madagascariensis*: corrected “risk” column from Near Threatened to Vulnerable.
- *Scoteanax rueppellii*: removed from “cave_use” because no record available on cave use (Churchill, 2008).
- *Tadarida latouchei*: corrected “risk” column from Near Threatened to Endangered.
- *Taphozous hildegardeae*: corrected “risk” column from Vulnerable to Endangered.
- *Tylonycteris pygmaeus*: removed from “cave_use” because no record available on cave use.
- *Tylonycteris robustula*: removed from “cave_use” because no record available on cave use.
- *Vampyressa pusilla*: removed from “cave_use” because no record available on cave use.
- *Vespadelus pumilus*: removed from “cave_use” because no record available on cave use.
- *Vespertilio murinus*: corrected “family” column from Phyllostomidae to Vespertilionidae.

There were a number of species that are recorded to roost in caves based on Tanalgo et al. (2022)’s inclusion criteria that were not present in their Appendix 1 database:

- *Barbastella darjelingensis*
- *Carollia castanea* (https://www.gbif.org/species/2433178)
- *Chaerephon bivittatus* (IUCN, 2022)
- *Chaerephon major* (IUCN, 2022)
- *Chaerephon nigeriae* (https://www.gbif.org/species/5218711)
- *Chalinolobus neocaledonicus* (IUCN, 2022)
- *Chalinolobus picatus* (Churchill, 2008)
- *Cistugo lesueuri* (Watson, 1998)
- *Dermanura azteca* (https://www.gbif.org/species/2433261)
- *Dermanura bogotensis* (Pérez-Torres et al., 2015)
- *Dobsonia magna* (IUCN, 2022)
- *Eptesicus anatolicus* (https://www.gbif.org/pt/species/5787592)
- *Eptesicus bobrinskoi* (Benda and Reiter, 2006)
- *Eptesicus bottae* (IUCN, 2022)
- *Eptesicus lobatus* (IUCN, 2022)
- *Eptesicus ognevi* (IUCN, 2022)
- *Eptesicus platyops* (IUCN, 2022)
- *Euderma maculatum* (IUCN, 2022)
- *Eumops floridanus* (IUCN, 2022)
- *Glischropus tylopus* (IUCN, 2022)
- *Harpiola isodon* (IUCN, 2022)
- *Hipposideros doriae* (Wilson and Mittermeier, 2019c)
- *Hipposideros gentilis* (IUCN, 2022)
- *Hipposideros inexpectatus* (IUCN, 2022)
- *Hipposideros nicobarulae* (Aul et al., 2014)
- *Histiotus macrotus* (IUCN, 2022)
- *Hypsugo ariel* (IUCN, 2022)
- *Lonchophylla bokermanni* (Wilson and Mittermeier, 2019a)
- *Lonchophylla fornicata* (IUCN, 2022)
- *Lonchophylla orienticollina* (Wilson and Mittermeier, 2019a)
- *Lonchophylla peracchii* (Teixeira et al., 2013)
- *Micronycteris sanborni* (Feijó et al., 2015)
- *Miniopterus ambohitrensis* (IUCN, 2022)
- *Miniopterus egeri* (Wilson and Mittermeier, 2019b)
- *Miniopterus griveaudi* (Goodman et al., 2010)
- *Miniopterus manavi* (Nowak and Walker, 1994)
- *Miniopterus orianae* (Codd et al., 2003)
- *Miniopterus paululus* (IUCN, 2022)
- *Miniopterus robustior* (IUCN, 2022)
- *Mormopterus kalinowskii* (Flores et al., 2019)
- *Molossus currentium* (Gardner et al., 1970)
- *Molossus rufus* (https://ppbio.inpa.gov.br/en/Bat_Library/Species/Molossus_II)
- *Mops condylurus* (Monadjem et al., 2020)
- *Mormopterus kalinowskii* (IUCN, 2022)
- *Murina bicolor* (IUCN, 2022)
- *Myotis aelleni* (Goodman et al., 2009)
- *Myotis albescens* (Braun et al., 2009)
- *Myotis alcathoe* (IUCN, 2022)
- *Myotis ater* (https://www.gbif.org/species/168816085)
- *Myotis chiloensis* (Lilley et al., 2020)
- *Myotis longicaudatus* (IUCN, 2022)
- *Myotis occultus* (IUCN, 2022)
- *Myotis planiceps* (IUCN, 2022)
- *Myotis rufopictus* (https://www.gbif.org/species/5787575)
- *Myotis secundus* (IUCN, 2022)
- *Myotis sicarius* (IUCN, 2022)
- *Nyctalus lasiopterus* (Estók, 2007)
- *Pipistrellus endoi* (Jo et al., 2018)
- *Platymops setiger* (IUCN, 2022)
- *Platyrrhinus aurarius* (Wilson and Mittermeier, 2019a)
- *Plecotus kozlovi* (IUCN, 2022)
- *Plecotus wardi* (IUCN, 2022)
- *Rhinolophus belligerator* (Patrick et al., 2013)
- *Rhinolophus inops* (https://www.gbif.org/species/2432648)
- *Rhinolophus mabuensis* (IUCN, 2022)
- *Rhinolophus nereis* (https://www.gbif.org/species/2432635)
- *Rhinolophus robertsi* (Woinarski et al., 2014)
- *Rhinolophus thailandensis* (IUCN, 2022)
- *Rhinolophus xinanzhongguoensis* (Zhou et al., 2009)
- *Saccopteryx antioquensis* (IUCN, 2022)
- *Sauromys petrophilus* (IUCN, 2022)
- *Tadarida lobata* (IUCN, 2022)
- *Taphozous mauritianus* (Dengis, 1996)
- *Trinycteris nicefori* (Cubero Vásquez and Artavia Durán, 2017)
- *Xeronycteris vieirai* (Dias and de Oliveira, 2020)

Additionally, the following species from Tanalgo et al. (2022) were not found in the IUCN Red List of Threatened Species database as of 10th November 2022 (IUCN, 2022), and therefore were excluded from the dataset:

- *Aselliscus dongbacana*
- *Hipposideros inexpectus*
- *Murina fanjingshanensis*
- *Nyctophilus howensis* (extinct)
- *Plecotus gaisleri*
- *Rhinolophus monocerus*
- *Tylonycteris fulvida*
- *Tylonycteris tonkinensis*

Lastly, there were also a number of duplicated species name from Tanalgo et al. (2022) that were removed.

# SUPPLEMENTARY TABLES

**Table S1.** **Cave-roosting bat species occurring in the southern hemisphere at risk of *Pseudogymnoascus destructans* (*Pd*) exposure**. Species were listed when >5% of their distribution overlaps *Pd* growth potential for the mean annual surface temperature (MAST) and cave depth-adjusted MAST. % range represent percentage of range overlap that each species has with roost temperatures predicted from the mean annual surface temperature within the range of 0–19.8 °C for growth of the fungal pathogen causing white-nose syndrome. Hib. studied represents whether winter hibernation has been studied in free-ranging bats and published in the literature. References for hibernation studies are provided in the raw data file. IUCN Threatened status was abbreviated where DD = data deficient, LC = least concern, NT = near threatened, VU = vulnerable, EN = endangered, CR = critically endangered.

| **Region** | **Family** | **Species** | **% range (MAST)** | **% range. (cave depth-adjusted MAST)** | **Hib. Studies** | **Threat status** |
| --- | --- | --- | --- | --- | --- | --- |
| Africa | Cistugidae | *Cistugo lesueuri* | 100 | 100 |  | LC |
| Africa | Molossidae | *Mormopterus francoismoutoui* | 100 | 100 |  | LC |
| Africa | Rhinolophidae | *Rhinolophus capensis* | 99.73 | 100 |  | LC |
| Africa | Cistugidae | *Cistugo seabrae* | 72.06 | 85.81 |  | LC |
| Africa | Rhinolophidae | *Rhinolophus darlingi* | 56.21 | 62.73 |  | LC |
| Africa | Vespertilionidae | *Eptesicus hottentotus* | 50.35 | 67.71 |  | LC |
| Africa | Miniopteridae | *Miniopterus sororculus* | 50.00 | 58.20 |  | LC |
| Africa | Rhinolophidae | *Rhinolophus cohenae* | 50.00 | 58.33 |  | VU |
| Africa | Rhinolophidae | *Rhinolophus damarensis* | 49.15 | 63.86 |  | LC |
| Africa | Hipposideridae | *Cloeotis percivali* | 45.59 | 60.92 |  | LC |
| Africa | Rhinolophidae | *Rhinolophus smithersi* | 44.25 | 54.87 |  | NT |
| Africa | Rhinolophidae | *Rhinolophus ruwenzorii* | 42.86 | 45.71 |  | EN |
| Africa | Rhinolophidae | *Rhinolophus clivosus* | 41.63 | 47.63 |  | LC |
| Africa | Hipposideridae | *Hipposideros caffer* | 33.75 | 42.41 |  | LC |
| Africa | Miniopteridae | *Miniopterus natalensis* | 32.03 | 44.01 |  | LC |
| Africa | Rhinolophidae | *Rhinolophus swinnyi* | 30.97 | 44.51 |  | LC |
| Africa | Hipposideridae | *Hipposideros megalotis* | 30.31 | 35.53 |  | LC |
| Africa | Molossidae | *Mormopterus jugularis* | 28.48 | 37.46 |  | LC |
| Africa | Rhinolophidae | *Rhinolophus simulator* | 27.52 | 36.12 |  | LC |
| Africa | Rhinolophidae | *Rhinolophus denti* | 27.08 | 46.51 |  | LC |
| Africa | Miniopteridae | *Miniopterus fraterculus* | 26.73 | 35.64 |  | LC |
| Africa | Rhinolophidae | *Rhinolophus maendeleo* | 25.00 | 25.00 |  | DD |
| Africa | Rhinolophidae | *Rhinolophus willardi* | 25.00 | 25.00 |  | EN |
| Africa | Miniopteridae | *Miniopterus majori* | 24.64 | 32.75 |  | LC |
| Africa | Vespertilionidae | *Myotis tricolor* | 23.88 | 31.85 |  | LC |
| Africa | Molossidae | *Platymops setiger* | 23.45 | 27.24 |  | LC |
| Africa | Molossidae | *Chaerephon bivittatus* | 22.19 | 29.67 |  | LC |
| Africa | Molossidae | *Chaerephon bemmeleni* | 21.10 | 27.64 |  | LC |
| Africa | Miniopteridae | *Miniopterus africanus* | 17.13 | 21.42 |  | DD |
| Africa | Vespertilionidae | *Myotis goudoti* | 16.62 | 22.69 |  | LC |
| Africa | Miniopteridae | *Miniopterus manavi* | 16.50 | 22.53 |  | LC |
| Africa | Molossidae | *Chaerephon atsinanana* | 16.30 | 28.26 |  | LC |
| Africa | Miniopteridae | *Miniopterus gleni* | 15.94 | 21.89 |  | LC |
| Africa | Emballonuridae | *Paremballonura atrata* | 15.86 | 24.83 |  | LC |
| Africa | Rhinolophidae | *Rhinolophus eloquens* | 15.55 | 19.11 |  | LC |
| Africa | Nycteridae | *Nycteris thebaica* | 14.76 | 20.18 | Yes | LC |
| Africa | Rhinolophidae | *Rhinolophus fumigatus* | 11.23 | 17.54 |  | LC |
| Africa | Molossidae | *Chaerephon ansorgei* | 11.03 | 17.13 |  | LC |
| Africa | Miniopteridae | *Miniopterus petersoni* | 10.00 | 10.00 |  | DD |
| Africa | Hipposideridae | *Macronycteris vittatus* | 9.37 | 15.86 |  | NT |
| Africa | Rhinolophidae | *Rhinolophus landeri* | 6.52 | 10.47 |  | LC |
| Africa | Rhinolophidae | *Rhinolophus mabuensis* | 6.45 | 6.45 |  | EN |
| Africa | Miniopteridae | *Miniopterus inflatus* | 5.38 | 6.55 |  | LC |
| Africa | Emballonuridae | *Coleura afra* | 5.01 | 6.29 |  | LC |
| Africa | Hipposideridae | *Hipposideros ruber* | 4.57 | 6.95 |  | LC |
| Africa | Emballonuridae | *Coleura kibomalandy* | 0.00 | 33.33 |  | DD |
| Asia/Australia | Miniopteridae | *Miniopterus australis* | 9.14 | 11.45 |  | LC |
| Asia/Australia | Hipposideridae | *Hipposideros diadema* | 5.48 | 6.76 |  | LC |
| Australia | Rhinolophidae | *Rhinolophus megaphyllus* | 43.86 | 46.51 | Yes | LC |
| Australia | Miniopteridae | *Miniopterus orianae* | 35.56 | 38.57 | Yes |  |
| Australia | Vespertilionidae | *Vespadelus troughtoni* | 33.73 | 39.57 |  | LC |
| Australia | Emballonuridae | *Taphozous troughtoni* | 3.27 | 5.97 |  | LC |
| Australia | Vespertilionidae | *Vespadelus finlaysoni* | 2.11 | 6.77 |  | LC |
| North America/South America | Phyllostomidae | *Lonchophylla robusta* | 21.12 | 23.86 |  | LC |
| North America/South America | Molossidae | *Nyctinomops macrotis* | 18.44 | 20.94 | Yes | LC |
| North America/South America | Phyllostomidae | *Diphylla ecaudata* | 13.12 | 15.78 |  | LC |
| North America/South America | Phyllostomidae | *Glossophaga soricina* | 11.82 | 13.88 |  | LC |
| North America/South America | Molossidae | *Nyctinomops laticaudatus* | 8.68 | 10.65 |  | LC |
| North America/South America | Phyllostomidae | *Carollia perspicillata* | 7.39 | 9.39 |  | LC |
| North America/South America | Phyllostomidae | *Macrophyllum macrophyllum* | 7.00 | 8.88 |  | LC |
| North America/South America | Emballonuridae | *Peropteryx kappleri* | 4.55 | 5.57 |  | LC |
| North America/South America | Emballonuridae | *Peropteryx macrotis* | 4.37 | 5.83 |  | LC |
| North America/South America | Phyllostomidae | *Lonchorhina aurita* | 3.90 | 5.42 |  | LC |
| Oceania | Hipposideridae | *Hipposideros corynophyllus* | 100 | 100 |  | LC |
| Oceania | Rhinolophidae | *Rhinolophus mcintyrei* | 35.62 | 39.73 |  | DD |
| Oceania | Miniopteridae | *Miniopterus macrocneme* | 24.59 | 27.05 |  | LC |
| Oceania | Emballonuridae | *Emballonura furax* | 24.39 | 29.27 |  | LC |
| Oceania | Emballonuridae | *Emballonura raffrayana* | 23.30 | 25.09 |  | LC |
| Oceania | Emballonuridae | *Emballonura beccarii* | 22.31 | 25.62 |  | LC |
| Oceania | Hipposideridae | *Hipposideros wollastoni* | 16.28 | 18.60 |  | LC |
| Oceania | Emballonuridae | *Emballonura dianae* | 6.09 | 9.57 |  | LC |
| South America | Molossidae | *Mormopterus phrudus* | 100 | 100 |  | VU |
| South America | Vespertilionidae | *Myotis aelleni* | 100 | 100 |  | DD |
| South America | Vespertilionidae | *Myotis chiloensis* | 99.33 | 99.89 |  | LC |
| South America | Vespertilionidae | *Histiotus macrotus* | 86.10 | 93.26 |  | LC |
| South America | Phyllostomidae | *Platalina genovensium* | 83.70 | 85.19 |  | NT |
| South America | Molossidae | *Tomopeas ravus* | 75.51 | 77.55 |  | EN |
| South America | Furipteridae | *Amorphochilus schnablii* | 63.33 | 65.00 |  | VU |
| South America | Phyllostomidae | *Anoura cultrata* | 45.75 | 50.81 |  | LC |
| South America | Vespertilionidae | *Myotis izecksohni* | 45.61 | 57.89 |  | DD |
| South America | Phyllostomidae | *Anoura fistulata* | 39.81 | 50.49 |  | DD |
| South America | Phyllostomidae | *Anoura latidens* | 35.39 | 40.48 |  | LC |
| South America | Phyllostomidae | *Artibeus fimbriatus* | 33.03 | 43.37 |  | LC |
| South America | Molossidae | *Nyctinomops aurispinosus* | 20.23 | 25.26 |  | LC |
| South America | Phyllostomidae | *Anoura geoffroyi* | 16.52 | 19.38 |  | LC |
| South America | Phyllostomidae | *Lonchophylla handleyi* | 14.53 | 17.09 |  | LC |
| South America | Phyllostomidae | *Anoura caudifer* | 12.50 | 15.54 |  | LC |
| South America | Molossidae | *Molossops temminckii* | 10.64 | 13.39 |  | LC |
| South America | Phyllostomidae | *Lionycteris spurrelli* | 5.22 | 5.80 |  | LC |

# SUPPLEMENTARY FIGURES


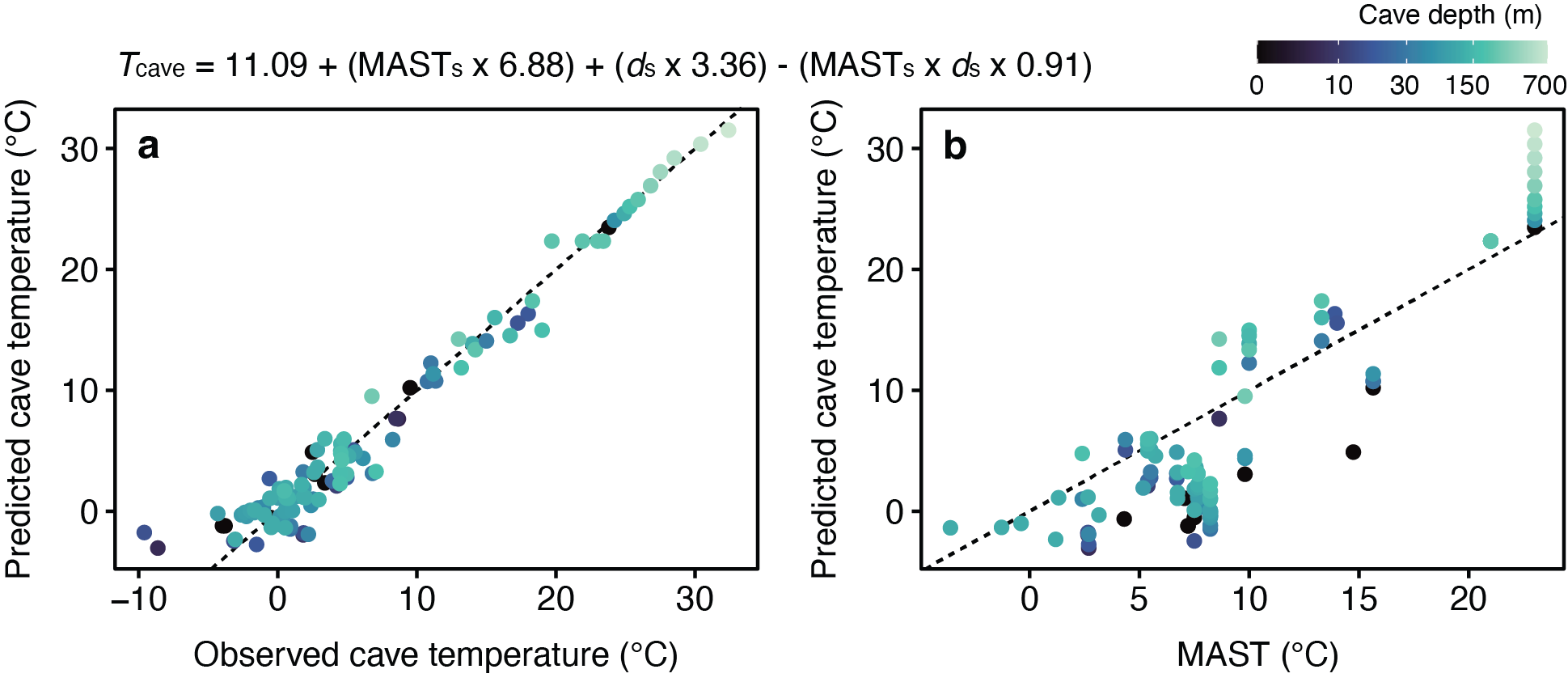


**Fig. S1. The predicted winter cave temperature (°C) against (a) the observed winter cave temperature (°C) and (b) the mean annual surface temperature (°C).** The coloured points represent distance from the cave entrance where the cave temperature was recorded. The dashed line represents a 1:1 scale relationship. The interaction of MAST and cave depth provided a more accurate estimate of cave temperatures (*R*^2^ = 0.931), over MAST alone (*R*^2^ = 0.881). Equation to convert mean annual surface temperature and cave depth to cave temperature presented on the top of the graph. *T*_cave_ represents the cave temperature, MAST_s_ is the standardised MAST, and *d*_s_ is the standardised cave depth.

**Fig. S2. Probability of Eurasian host bat species developing WNS as a function of phylogenetic distance between species**. The main effect (curved line) is predicted from logistic regressions using coefficients in millions of years (my).


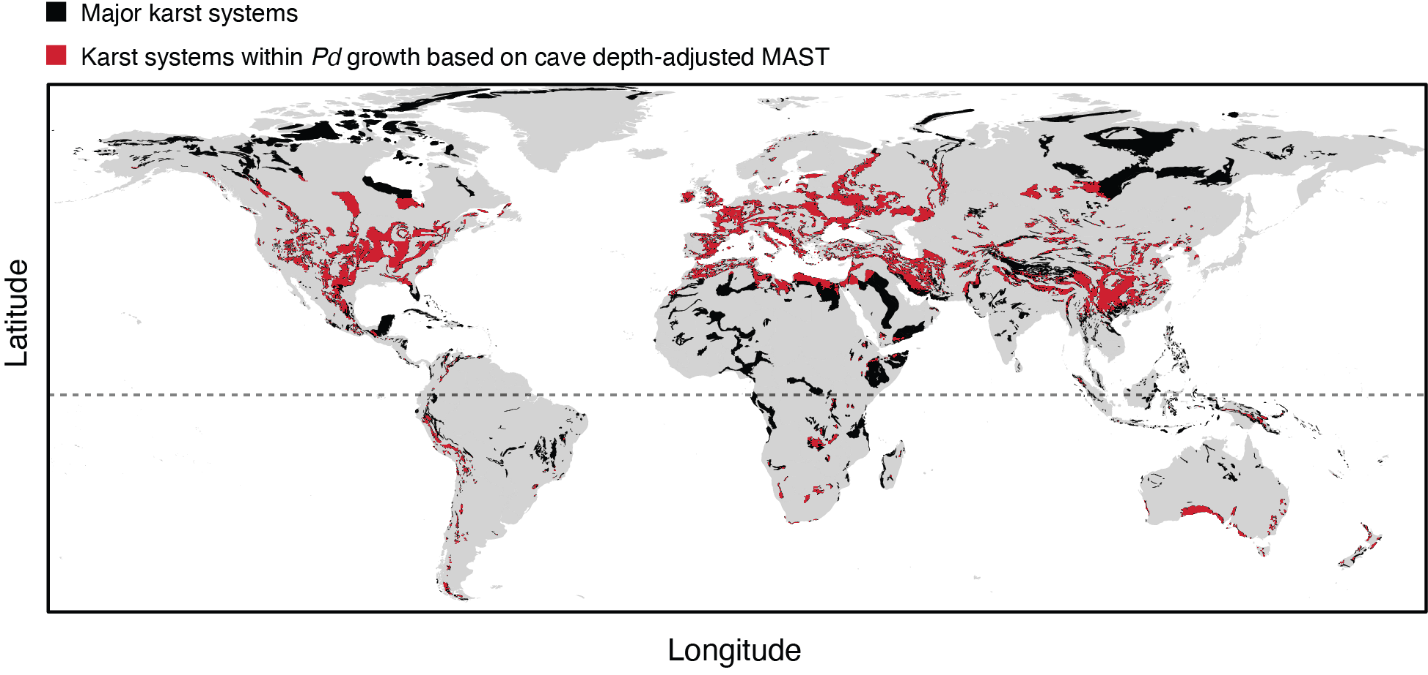


**Fig. S3. Spatial risk of cave-roosting bats across karst cave systems.** Black filled represent major karst systems, red filled areas represent karst systems with temperatures, based on the cave depth-adjusted mean annual surface temperature (MAST) suitable for *Pd* growth. Note, cave-roosting caves are also known to roost in man-made structures (mines, culverts bridges). Karst caves were obtained from the World-wide Hydrogeological Mapping and Assessment Programme World Karst Aquifer Map project (<https://produktcenter.bgr.de/terraCatalog/OpenSearch.do?search=ab3b15cb-a6c3-42ea-ae0c-0b417d698949&type=/Query/OpenSearch.do>).


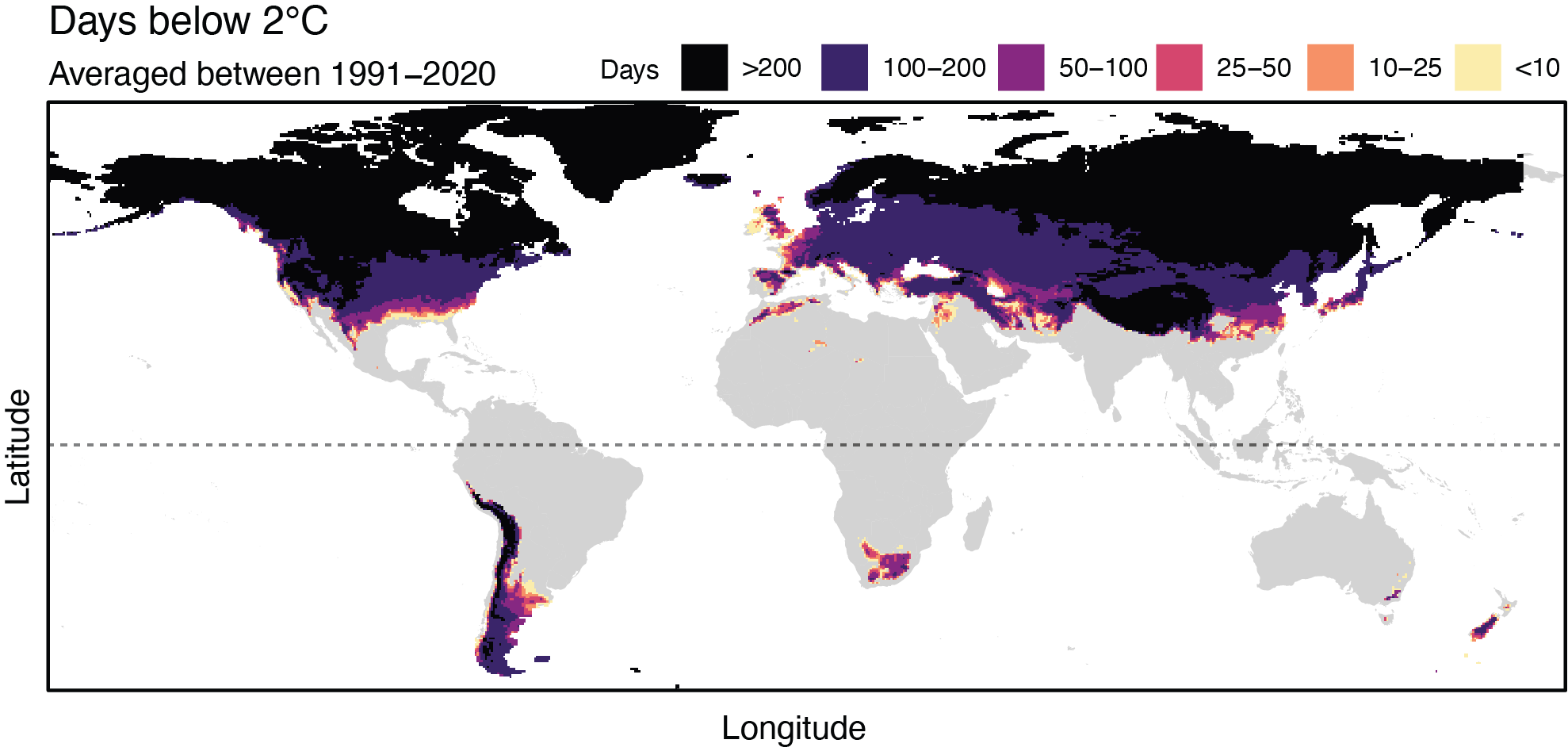


**Fig. S4. Potential frost days based on ambient air temperature below 2°C**. Frost formation depends on the temperature, cloud coverage, relative humidity, surface winds, and topography. However, temperature is generally used as a broader-scale indicator of frost in the absence of detailed information of other environmental variables. An observed temperature of 2.2°C at the instrument level (1.2 m above ground) indicates that the temperature at the surface level is approaching 0°C. The daily minimum air temperature was extracted from the CPC Global Unified Temperature provided by the NOAA PSL, Boulder, Colorado, USA.


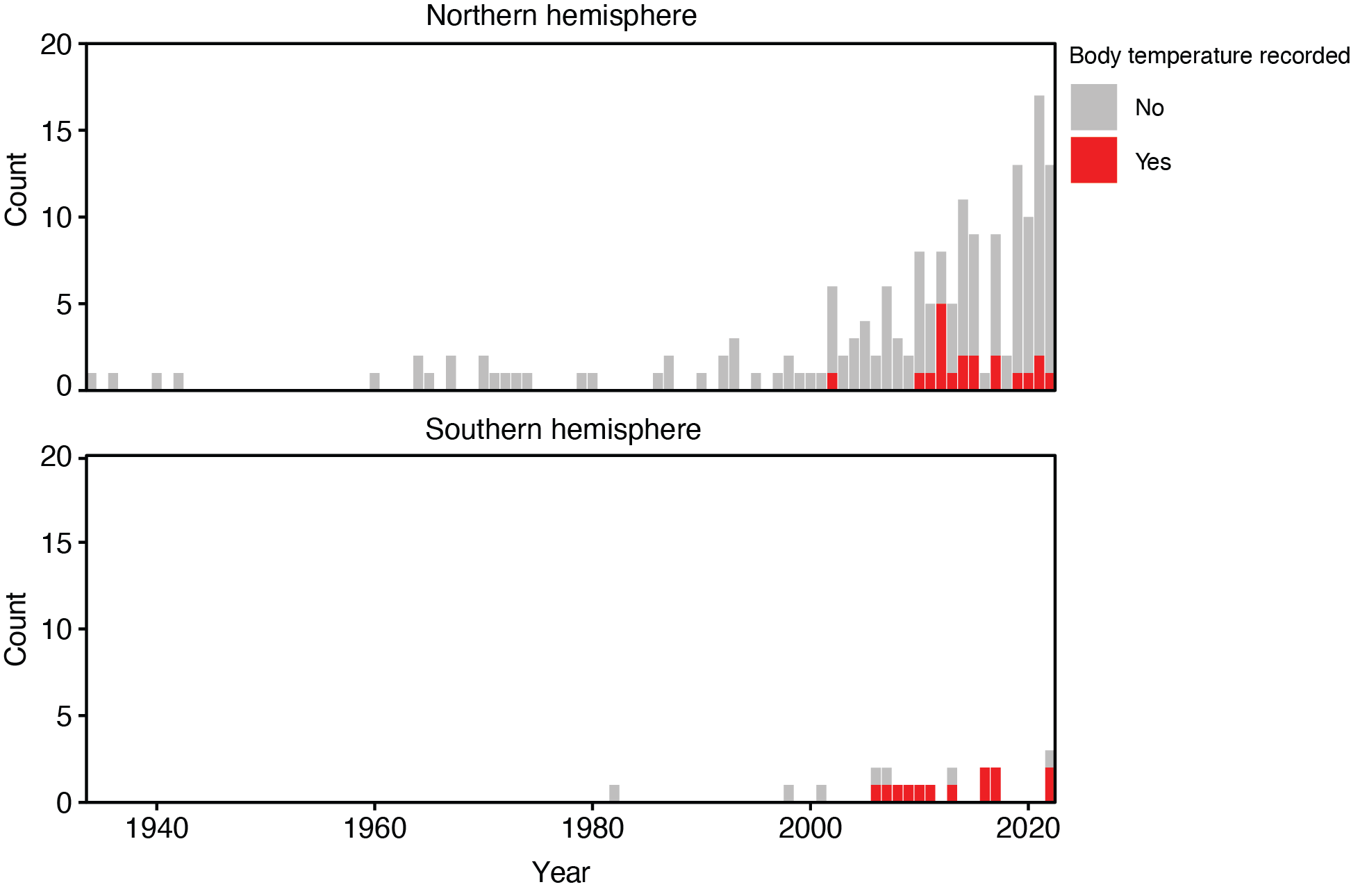


**Fig. S5. Number of primary peer-reviewed studies examining hibernation or torpor during winter for free-living bats.** Top graph shows number of studies conducted in countries from the northern hemisphere (173 studies), and the bottom graph shows the number of studies conducted in countries from the southern hemisphere (20 studies). Red counts represent studies that measured body temperature of free-ranging bats during winter, while the grey counts represent other methods that provide information on hibernation behaviour of bats in the wild (acoustic monitoring, cave survey, banding, camera trap, collection, PIT-tagging).

# SUPPLEMENTARY REFERENCES

**Aul, B., Bates, P., Harrison, D. and Marimuthu, G.** (2014). Diversity, distribution and status of bats on the Andaman and Nicobar Islands, India. *Oryx* **48**, 204-212.

**Benda, P. and Reiter, A.** (2006). On the occurrence of *Eptesicus bobrinskoi* in the Middle East (Chiroptera: Vespertilionidae). *Lynx* **37**, 23-44.

**Braun, J. K., Layman, Q. D. and Mares, M. A.** (2009). *Myotis albescens* (Chiroptera: Vespertilionidae). *Mamm. Species*, 1-9.

**Bredt, A., Uieda, W. and Magalhães, E. D.** (1999). Cave bats from the Distrito Federal area in mid-western Brazil (Mammalia, Chiroptera). *Rev. Bras. Zool.* **16**, 731-770.

**Churchill, S.** (2008). Australian bats. Crows Nest, Australia: Allen & Unwin.

**Codd, J. R., Sanderson, K. J. and Branford, A. J.** (2003). Roosting activity budget of the southern bent-wing bat (*Miniopterus schreibersii bassanii*). *Aust. J. Zool.* **51**, 307-316.

**Cubero Vásquez, O. and Artavia Durán, E.** (2017). First record of the bat T*rinycteris nicefori* (Chiroptera: Phyllostomidae) in the Nicoya Peninsula, Guanacaste, Costa Rica. *Cuadernos de Investigación UNED* **9**, 35-37.

**Dengis, C. A.** (1996). *Taphozous mauritianus*. *Mamm. Species*, 1-5.

**Dias, D. and de Oliveira, M. B.** (2020). First record of *Xeronycteris vieirai* Gregorin & Ditchfield, 2005 (Chiroptera, Phyllostomidae) for the Cerrado biome. *Oecologia Australis* **24**, 696-703.

**Estók, P.** (2007). Seasonal changes in the sex ratio of Nyctalus species in north-east Hungary. *Acta Zool. Acad. Sci. Hung.* **53**, 89-95.

**Feijó, A., Rocha, P. A.-d. and Ferrari, S. F.** (2015). How do we identify *Micronycteris* (*Schizonycetris*) *sanborni* Simmons, 1996 (Chiroptera, Phyllostomidae) reliably and where we can find this species in Brazil? *Papéis Avulsos de Zoologia* **55**, 269-280.

**Flores, M., Calizaya, G., Portugal-Zegarra, G., Aragón, G., Pacheco-Castillo, J. and Rengifo, E.** (2019). Contributions to the natural history of Mormopterus kalinowskii (Chiroptera: Molossidae) in the southwest of Peru Therya Advance. *Associación Mexicana de Mastozoología* **10**, 1-10.

**Gardner, A. L., LaVal, R. K. and Wilson, D. E.** (1970). The distributional status of some Costa Rican bats. *J. Mammal.* **51**, 712-729.

**Goodman, S. M., Weyeneth, N., Ibrahim, Y., Saïd, I. and Ruedi, M.** (2010). A review of the bat fauna of the Comoro Archipelago. *Acta Chiropt.* **12**, 117-141.

**Goodman, S. M., Maminirina, C. P., Weyeneth, N., Bradman, H. M., Christidis, L., Ruedi, M. and Appleton, B.** (2009). The use of molecular and morphological characters to resolve the taxonomic identity of cryptic species: the case of *Miniopterus manavi* (Chiroptera, Miniopteridae). *Zool. Scr.* **38**, 339-363.

**IUCN.** (2022). The IUCN Red List of threatened species. Version 2022-2. <<https://www.iucnredlist.org/>>

**Jo, Y.-S., Baccus, J. T. and Koprowski, J. L.** (2018). Mammals of Korea: a review of their taxonomy, distribution and conservation status. *Zootaxa* **4522**, 1–216.

**Lilley, T. M., Sävilammi, T., Ossa, G., Blomberg, A. S., Vasemägi, A., Yung, V., Vendrami, D. L. and Johnson, J. S.** (2020). Population connectivity predicts vulnerability to white-nose syndrome in the Chilean myotis (*Myotis chiloensis*)-a genomics approach. *G3: Genes, Genomes, Genetics* **10**, 2117-2126.

**Molur, S., Marimuthu, G., Srinivasulu, C., Mistry, S., Hutson, A. M., Bates, P. J., Walker, S., Priya, K. P. and Priya, A. B.** (2002). Status of South Asian Chiroptera. In *Conservation Action Management Plan (CAMP) Workshop Report, Zoo Outreach Organisation, 320pp*.

**Monadjem, A., Taylor, P. J. and Schoeman, M. C.** (2020). Bats of southern and central Africa: a biogeographic and taxonomic synthesis: Wits University Press.

**Nowak, R. M. and Walker, E. P.** (1994). Walker's bats of the world. Maryland, US: Johns Hopkins University Press.

**Patrick, L. E., McCulloch, E. S. and Ruedas, L. A.** (2013). Systematics and biogeography of the arcuate horseshoe bat species complex (Chiroptera, Rhinolophidae). *Zool. Scr.* **42**, 553-590.

**Pérez-Torres, J., Martínez-Medina, D., Peñuela-Salgado, M., Ríos-Blanco, M. C., Estrada-Villegas, S. and Martínez-Luque, L.** (2015). Macaregua: the cave with the highest bat richness in Colombia. *Check List* **11**, 1616-1616.

**Richards, G. C.** (1995). White-striped freetail-bat *Nyctinomus australis* (Gray, 1838). In *The Mammals of Australia*, (ed. R. Strahan), pp. 487-488. Chatswood, NSW: Reed Books.

**Seltmann, A., Czirják, G. Á., Courtiol, A., Bernard, H., Struebig, M. J. and Voigt, C. C.** (2017). Habitat disturbance results in chronic stress and impaired health status in forest-dwelling paleotropical bats. *Conserv. Physiol.* **5**.

**Smith, A. T., Xie, Y., Hoffmann, R. S., Lunde, D., MacKinnon, J., Wilson, D. E., Wozencraft, W. C. and Gemma, F.** (2010). A guide to the mammals of China. Oxfordshire, UK: Princeton University Press.

**Tanalgo, K. C., Oliveira, H. F. and Hughes, A. C.** (2022). Mapping global conservation priorities and habitat vulnerabilities for cave-dwelling bats in a changing world. *Sci. Total Environ.* **843**, 156909.

**Teixeira, T. S. M., Rosa, D. T. C., Dias, D., Cerqueira, R. and Vale, M. M.** (2013). First record of *Lonchophylla peracchii* Dias, Esbérard and Moratelli, 2013 (Chiroptera, Phyllostomidae) in São Paulo State, Southeastern Brasil. *Oecologia Australis* **17**, 424-428.

**Watson, J.** (1998). New distributional records for three microchiropteran bats (Vespertillionidae, Rhinolophidae) from the Free State Province, South Africa. *S. Afr. J. Wildl. Res.* **28**, 127-131.

**Wilson, D. E. and Mittermeier, R. A.** (2019a). Phyllostomidae. In *Handbook of the Mammals of the World*, vol. 9 Bats, pp. 444-583: Lynx Edicions.

**Wilson, D. E. and Mittermeier, R. A.** (2019b). Miniopteridae. In *Handbook of the Mammals of the World*, vol. 9 Bats, pp. 674-709: Lynx Edicions.

**Wilson, D. E. and Mittermeier, R. A.** (2019c). Hipposideridae. In *Handbook of the Mammals of the World*, vol. 9 Bats, pp. 227-258 Lynx Edicions.

**Woinarski, J. C., Burbidge, A. and Harrison, P.** (2014). The action plan for Australian mammals 2012. Collingwood, Australia: CSIRO publishing.

**Zhou, Z.-M., Guillén-Servent, A., Lim, B. K., Eger, J. L., Wang, Y.-X. and Jiang, X.-L.** (2009). A new species from southwestern China in the Afro-Palearctic lineage of the horseshoe bats (*Rhinolophus*). *J. Mammal.* **90**, 57-73.
